# *Madrepora oculata* forms large frameworks in hypoxic waters off Angola (SE Atlantic)

**DOI:** 10.1038/s41598-021-94579-6

**Published:** 2021-07-26

**Authors:** Covadonga Orejas, Claudia Wienberg, Jürgen Titschack, Leonardo Tamborrino, André Freiwald, Dierk Hebbeln

**Affiliations:** 1 Instituto Español de Oceanografía, Centro Oceanográfico de Gijón (IEO, CSIC), Avenida Príncipe de Asturias 70 bis, 33212 Gijón, Spain; 2 MARUM-Center for Marine Environmental Sciences, University of Bremen, Leobener Str. 8, 28359 Bremen, Germany; 3 Senckenberg am Meer, Südstrand 40, 26382 Wilhelmshaven, Germany

**Keywords:** Coral reefs, Biogeography

## Abstract

This study aims to map the occurrence and distribution of *Madrepora oculata* and to quantify density and colony sizes across recently discovered coral mounds off Angola. Despite the fact that the Angolan populations of *M. oculata* thrive under extreme hypoxic conditions within the local oxygen minimum zone, they reveal colonies with remarkable heights of up to 1250 mm—which are the tallest colonies ever recorded for this species—and average densities of 0.53 ± 0.37 (SD) colonies m^−2^. This is particularly noteworthy as these values are comparable to those documented in areas without any oxygen constraints. The results of this study show that the distribution pattern documented for *M. oculata* appear to be linked to the specific regional environmental conditions off Angola, which have been recorded in the direct vicinity of the thriving coral community. Additionally, an estimated average colony age of 95 ± 76 (SD) years (total estimated age range: 16–369 years) indicates relatively old *M. oculata* populations colonizing the Angolan coral mounds. Finally, the characteristics of the Angolan populations are benchmarked and discussed in the light of the existing knowledge on *M. oculata* gained from the North Atlantic and Mediterranean Sea.

## Introduction

**Figure 1:**
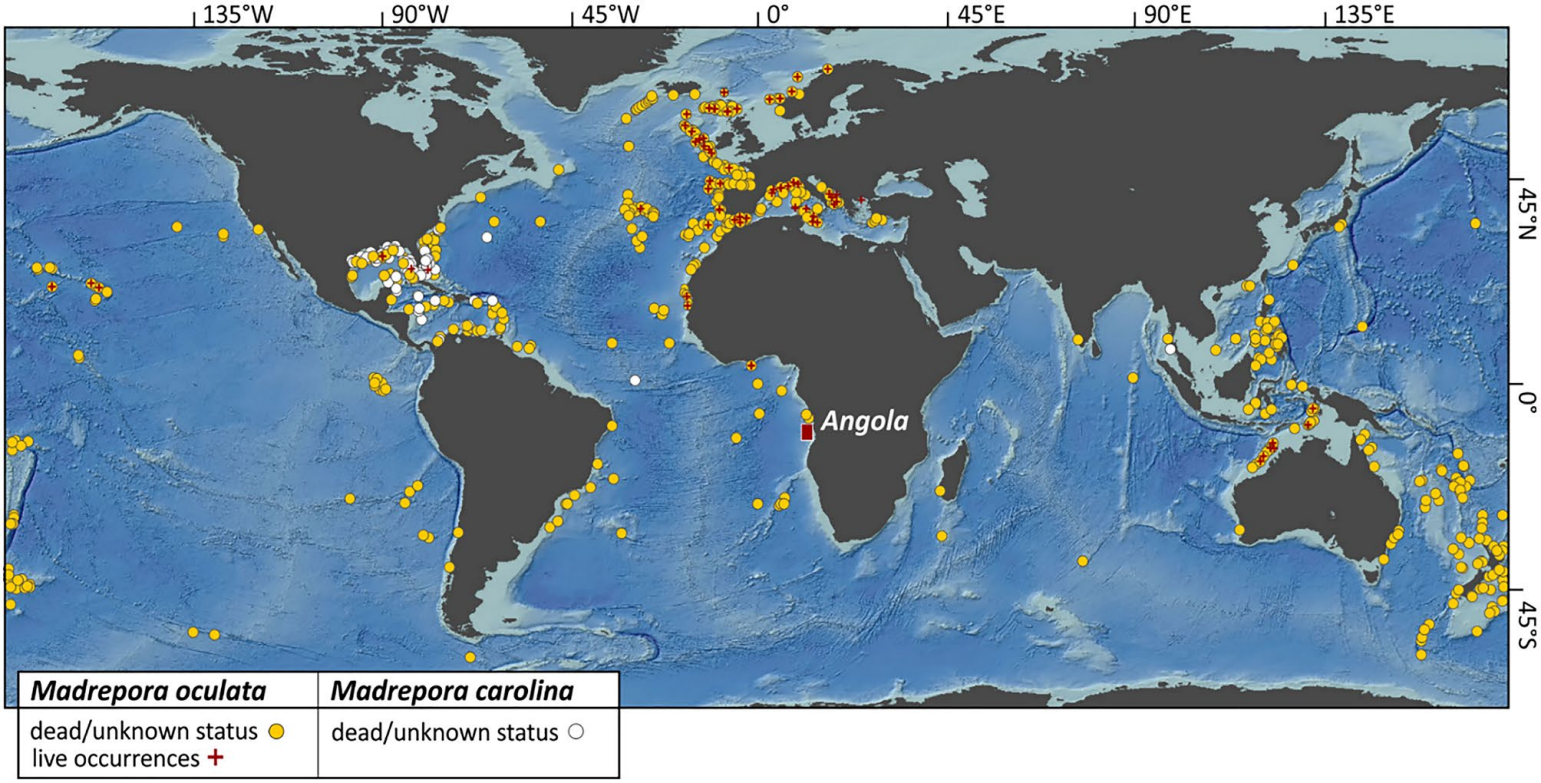
Geographical distribution of *Madrepora oculata* and *Madrepora carolina*. The global map shows the distribution of the two reef-forming species of the genus *Madrepora* (sources: World Conservation Monitoring Centre of the United Nation Environmental Programme, UNEP-WCMC; Ocean Biogeographic Information System, OBIS). While a large number of records of *M. oculata* (yellow dots; live occurrences indicated by a red cross) is reported in the North Atlantic and the West and South Pacific, *M. carolina* (white dots) is endemic to the NW Atlantic. The red polygon highlights the study area off Angola in the SW Atlantic, where living *M. oculata* have been discovered for the first time. The map is based on the digital elevation model (DEM) CleanTOPO2 (an edited version of the SRTM30 Plus) published by Patterson (2006; http://www.shadedrelief.com/cleantopo2) and was created with ESRI ArcGIS Desktop 10.7.

The most ubiquitous and conspicuous reef-forming cold-water coral (CWC) species in the Atlantic Ocean are *Lophelia pertusa* and *Madrepora oculata*^1^. For both species, information is available regarding their occurrence, distribution and physiological performance, and a lot of advances have been made to improve the understanding of their biology, ecology and environmental constraints in present and future scenarios^2,3^. However, the number of published studies addressing CWCs is largely biased towards *L. pertusa*^4^. Furthermore, most of the gathered knowledge on *M. oculata* is rather punctual and restricted to specific locations in the NE Atlantic^4,5^ and the Mediterranean Sea^6,7^. Despite the fact that the current research on *M. oculata* is locally limited, it is considered a cosmopolitan species just like *L. pertusa*. It has an extended geographical distribution ranging from northern Norway (Korallen Reef) at 70° N, 22° E^8^ to the sub-Antarctic (Drake Passage) at 60° S, 69° W^9^, shows a high abundance in the West (off Indonesia, Philippines and Japan^10^) and South Pacific (off New Zealand^11^), and has recently also been documented from the Central Pacific (Phoenix Islands Protected Area, Republic of Kirbati^12^). However, up until today regarding the entire Indian Ocean, limited records have been registered (Fig. 1). In some areas of the Mediterranean Sea, *M. oculata* dominates the CWC communities^6,7,13–16^ or even constitutes the only framework-building CWC^17^. In some areas of the NE Atlantic, it occurs in similar numbers as *L. pertusa*^4^. However, at most of the Atlantic sites explored so far, *L. pertusa* is by far the dominant reef-forming CWC species e.g.^18–22^.

Up to now, studies on the distribution pattern and population density of *M. oculata* have only been performed for few sites in the NE Atlantic and the Mediterranean Sea^4,7,15,23^. The northernmost investigated locations are the Norwegian CWC reefs (specifically the Røst Reef), for which maximum colony densities of 0.06 patches m^−2^ have been reported^23^. Overall, the highest average values for population density are documented for the NE Atlantic sites (Iceland, Ireland, Bay of Biscay) with values ranging from 0.53 ± 0.61 (SD) colonies m^−2^ to 1.64 ± 1.19 (SD) colonies m^−2^^4^, whereas *M. oculata* populations in the Mediterranean display average densities ranging from 0.11 ± 0.44 (SD) colonies m^−2^ (Cap de Creus canyon, Gulf of Lions^7^) to averages of 0.81 ± 1.87 (SD) colonies m^−2^ (Cabliers coral mound province, Alborán Sea^15^). In addition, although a more limited capacity to build large frameworks has been attributed to *M. oculata*, mostly due to the more fragile branches that the species displays^1^, some exceptions have been documented in the Mediterranean Sea, where remarkably large colonies of up to 1 m in height have recently been discovered in the Ligurian Sea^24^.

The physical–chemical properties of the ambient waters have a controlling influence on the physiology of *M. oculata* (and other CWCs) as revealed by various experimental studies. For example, *M. oculata* populations, thriving at temperatures of 12 °C in the Mediterranean Sea (Cap de Creus canyon), decrease their respiration and calcification rates when exposed to lowered temperatures^25^. Low pH conditions seem to have no effect on the calcification rate of *M. oculata* even after an exposure time of six months^26^. Studies on the influence of dissolved oxygen concentrations (DO) have only been conducted on *L. pertusa*^27,28^, providing diverging results. While *L. pertusa* populations from the NE Atlantic merely survive DO of > 3.3 mL L^−1^^27^, populations collected from the NW Atlantic can withstand oxygen levels as low as 1.6 mL L^−1^^28^. Additionally, a mixed assemblage of *L. pertusa* and *M. oculata* colonizing coral mounds off Mauritania—though in low numbers and spatially dispersed—are exposed to even lower DO of 1.0–1.3 mL L^−1^^29,30^.

In this work, we present the unique records of *M. oculata* observed on coral mounds off Angola, where hypoxic conditions with DO as low as 0.5 mL L^−1^ prevail^31,32^. The main aims of this study are to map the occurrence of *M. oculata* on the explored Angolan coral mounds, to quantify density and distribution of the species, and to discuss the observed distribution pattern in relation to the environmental setting in the region. Furthermore, demographic patterns are investigated, and based on the relations between colony size and growth rates obtained from other CWC areas, an average age of the Angolan *M. oculata* colonies is estimated. The results are discussed in the light of the existing knowledge on the species in the NE Atlantic and the Mediterranean Sea.

## Results

### Distribution and density patterns of the Angolan *Madrepora oculata* populations

**Figure 2:**
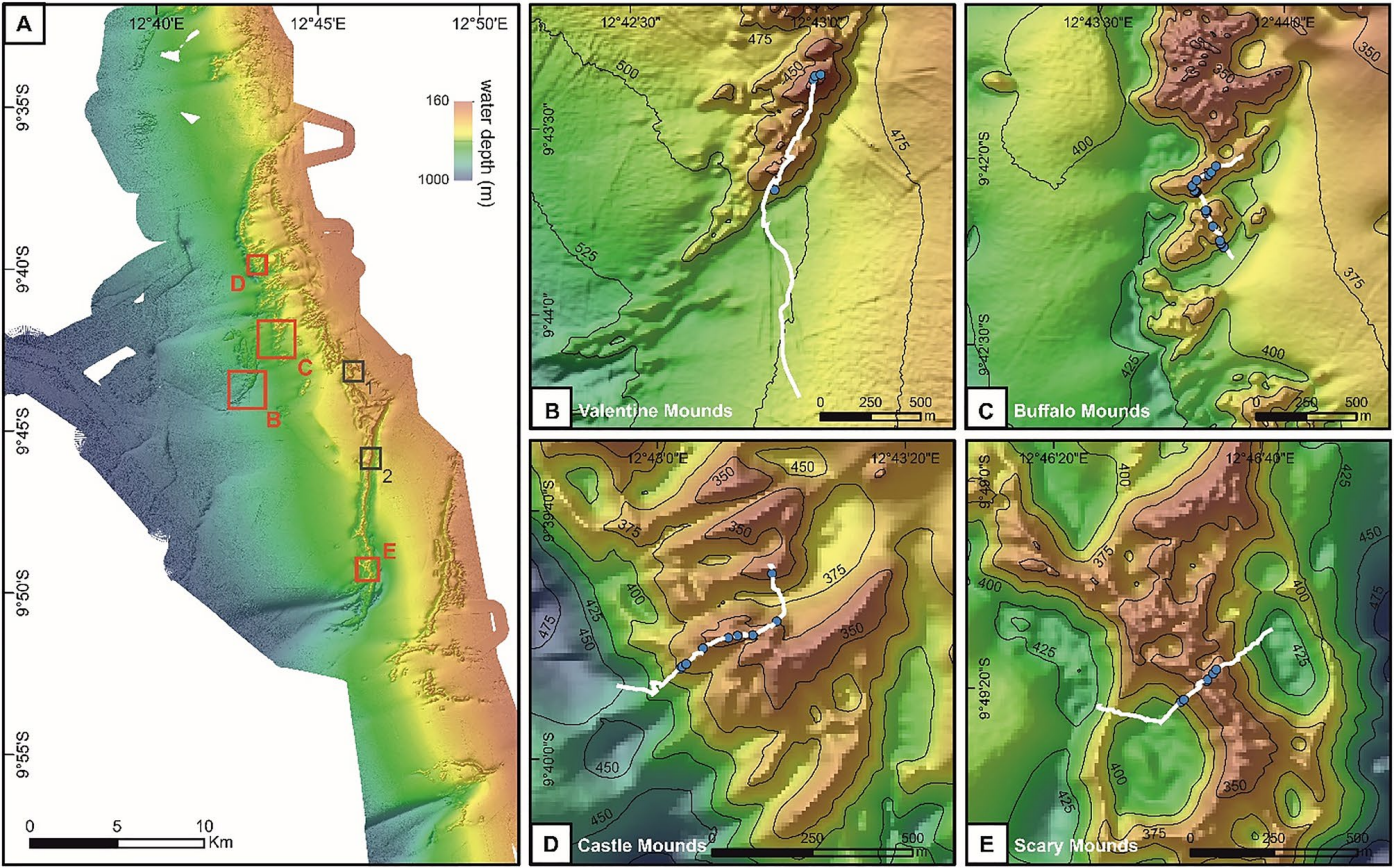
Occurrence of *Madrepora oculata* on the Angolan cold-water coral mounds. (**A**) Overview map of the Angolan coral mound province. Coral mounds for which ROV video footage was analysed are marked by boxes. Grey boxes indicate Snake mound (1) and Anna ridge (2), for which no *Madrepora* records have been documented; red boxes indicate the Valentine **(B)**, Buffalo **(C)**, Castle **(D)** and Scary **(E)** mounds being colonised by *M. oculata*. (**B–E**) Detailed maps of the Valentine, Buffalo, Castle and Scary coral mounds. ROV video transects (white line) crossing the respective mounds and identified locations of living *M. oculata* (blue dots) are indicated. Bathymetry data was acquired during R/V Meteror cruise M122 (Raw data is available under https://www2.bsh.de/daten/DOD/Bathymetrie/Suedatlantik/m122.htm).

*Madrepora oculata* was present on four of the six explored Angolan coral mounds (Buffalo, Castle, Scary, and Valentine mounds; Table 1), where a total of 83 colonies were discovered. The locations of these colonies were marked on high-resolution bathymetric maps (grid cell size: 10 m) providing detailed shaded-relief views of the mounds (Fig. 2b–e). The species was most abundant on Buffalo mound with 39 registered occurrences, followed by 30 on Castle, eight on Scary, and six on Valentine mound (Table 1). The overall average density of *M. oculata*-colonies including all coral mounds was 0.53 ± 0.37 (SD) colonies m^−2^, covering on average 1.25 ± 1.28 (SD) m^−2^ of the seafloor within the analyzed video frames (for details see Table 2).

**Table 1:** Metadata of seven ROV video surveys conducted across six Angolan coral mounds during RV Meteor cruise M122.

Coral mound	ROV transect	Date (dd.mm.yyyy)	Time (UTC)	Latitude	Longitude	Depth (m)	Total transect length (m)	Transect length covering the first and last Mo occurrence (m)	No. of video frames analysed	Area (m^2^) covered by the analysed video frames	No. live/dead Mo (no. measured Mo)
Anna ridge	Start	20.01.2016	10:00	9°44.76′ S	12°46.93′ E	336	856	0	0		0
	End	20.01.2016	16:19	9°44.61′ S	12°46.88′ E	251					
Snake	Start	23.01.2016	08:04	9°43.05′ S	12°45.86′ E	320	835	0	0		0
	End	23.01.2016	13:37	9°43.03′ S	12°46.10′ E	256					
Castle	Start	25.01.2016	06:05	9°39.90′ S	12°42.95′ E	447	796	255	7	51.2	30/0 (7)
	End	25.01.2016	12:45	9°39.74′ S	12°43.15′ E	331					
Scary	Start	22.01.2016	06:14	9°49.36′ S	12°46.41′ E	412	785	206	2	35.5	8/1 (2)
	End	22.01.2016	14:01	9°49.24′ S	12°46.69′ E	425					
Buffalo	Start	21.01.2016	06:56	9°42.27′ S	12°43.86′ E	402	951	492	7	49.2	39/3 (20)
	End	21.01.2016	14:42	9°42.00′ S	12°43.88′ E	356					
Valentine (a)	Start	19.01.2016	11:00	9°43.67′ S	12°42.89′ E	473	1,188	1,087	4	35.9	6/0 (1)
	End	19.01.2016	17:08	9°43.01′ S	12°43.01′ E	426					
Valentine (b)	Start	17.01.2016	09:15	9°43.77′ S	12°42.85′ E	502	948	0	0		0
	End	17.01.2016	16:30	9°43.66′ S	12°42.89′ E	483					

For the location of the ROV dives see Fig. 2. The number of live and dead occurrences of *Madrepora oculata* (No. live/dead Mo) as well as colonies for which size measurements were conducted (No. measured Mo; given in brackets) observed during the analyses of the down-look video frame are indicated for each dive.

**Table 2:** *Madrepora oculata* density and coverage on the Angolan coral mounds (R: range, Av.: average) analysed for 20 down-look video frames.

Coral mound	R colony density (colony m^−2^)	Av. colony density (colony m^−2^ ± SD)	R of area covered by video frames (m^2^)	Coral coverage (coverage m^−2^ ± SD)	No. video frames
Castle	0.27–0.83	0.55 ± 0.26	3.60–13.27	1.36 ± 1.16	7
Scary	0.15–0.27	0.21	13.36–22.10	0.29	2
Buffalo	0.15–1.43	0.72 ± 0.72	4.56–10.36	1.74 ± 1.67	7
Valentine	0.10–0.51	0.27 ± 0.22	3.91–10.16	0.69 ± 0.69	4
All	0.10–1.43	0.53 ± 0.37	3.60–22.10	1.25 ± 1.28	20

Even though *M. oculata* occurred over a total bathymetric range of 330–470 m water depth (Table 3), the majority of colonies (91%, recorded on Castle, Scary and Buffalo mounds) thrived in a very narrow depth range between 330 and 390 m depth. Only on Valentine mound, deep occurrences below 420 m were registered (Table 3). Consistently, *M. oculata* colonies could not be located on the shallow Anna ridge and Snake mound (250–330 m water depth).

**Table 3:** Depth range of living occurrence of *Madrepora oculata* (DR-LOM) recorded for each cold-water coral mound.

Coral mound	DR-LOM (m)	DR-CTD (m)	DO (mL L^−1^)		Temperature (°C)		Salinity	
			Min–max	Mean ± SD	Min–max	Mean ± SD	Min–max	Mean ± SD
Anna ridge	./.	251–336	0.7–0.9	0.8 ± 0.1	10.1–14.2	12.1 ± 1.2	34.9–35.4	35.2 ± 0.1
Snake	./.	256–320	0.7–0.8	0.7 ± 1.0	9.4–12.7	11.1 ± 1.0	34.9–35.3	35.1 ± 0.1
Castle	332–364	332–449	0.6–0.9	0.8 ± 0.6	7.8–9.3	8.4 ± 0.6	34.6–34.9	34.8 ± 0.1
Scary	357–382	335–428	0.5–0.7	0.7 ± 0.3	8.7–10.0	9.1 ± 0.3	34.7–34.9	34.8 ± 0.0
Buffalo	343–389	343–405	0.7–0.8	0.7 ± 1.0	9.1–12.2	10.3 ± 1.0	34.8–35.2	35.0 ± 0.1
Valentine (a)	416–472	413–474	0.7–1.3	0.9 ± 0.2	7.9–8.7	8.1 ± 0.2	34.7–34.8	34.7 ± 0.0
Valentine (b)	./.	483–502	0.9–1.3	1.03 ± 0.13	7.0–7.4	7.3 ± 0.1	34.6–34.7	34.6 ± 0.0

Additionally, the range and mean values of environmental parameters (DO: dissolved oxygen concentrations) recorded by a CTD installed on the ROV during video observations are provided (DR-CTD: depth range of CTD measurements)*.*

### *Madrepora* colony size structure

**Figure 3:**
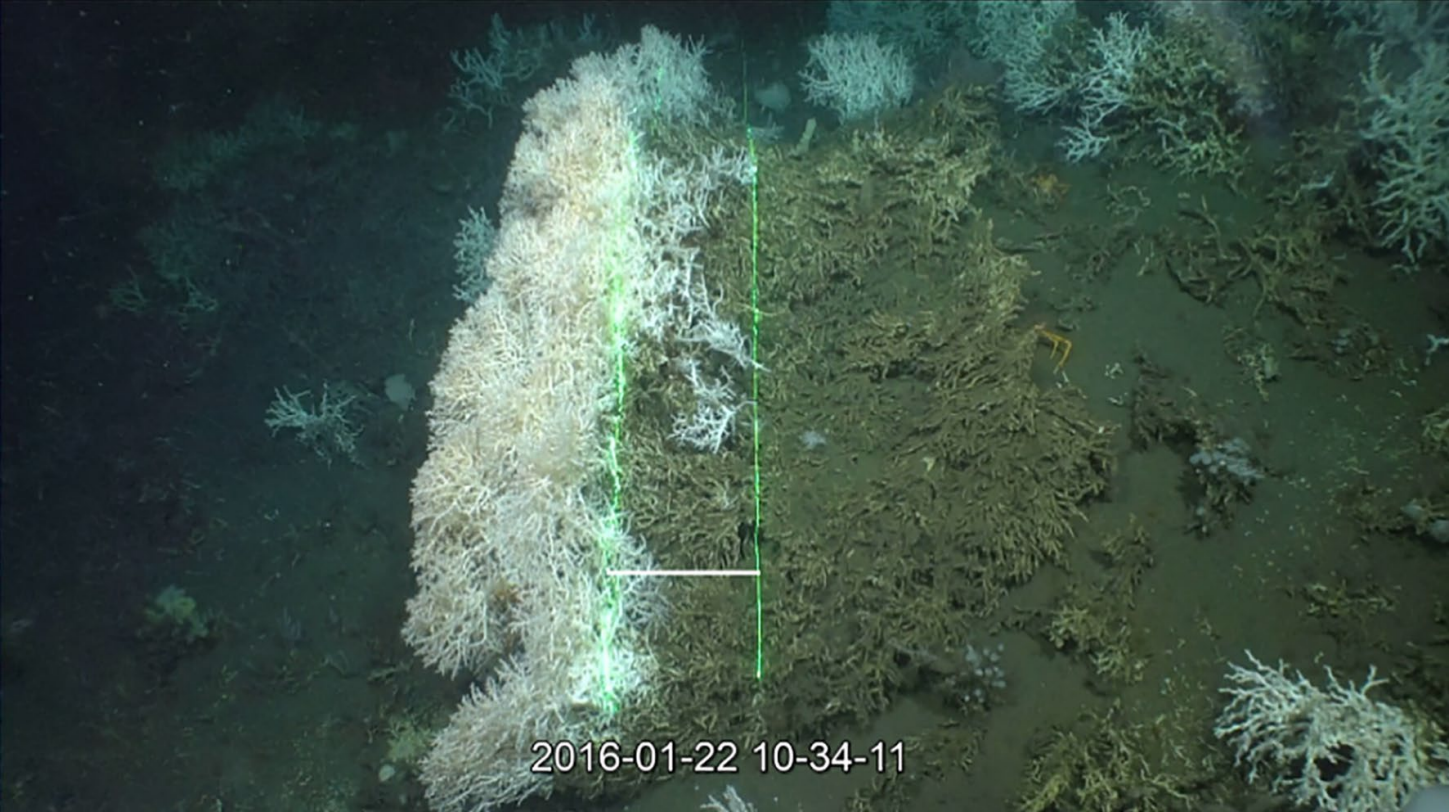
Scaling of large *Madrepora oculata* framework (Scary mound, Angola). The ROV used for video surveys was equipped with two line lasers. The parallel green lines are the projected laser beams, which are 30 cm apart (the distance between the beams is depicted by a white line) and are used as a scale. Credits: photograph by MARUM ROV SQUID.

**Figure 4:**
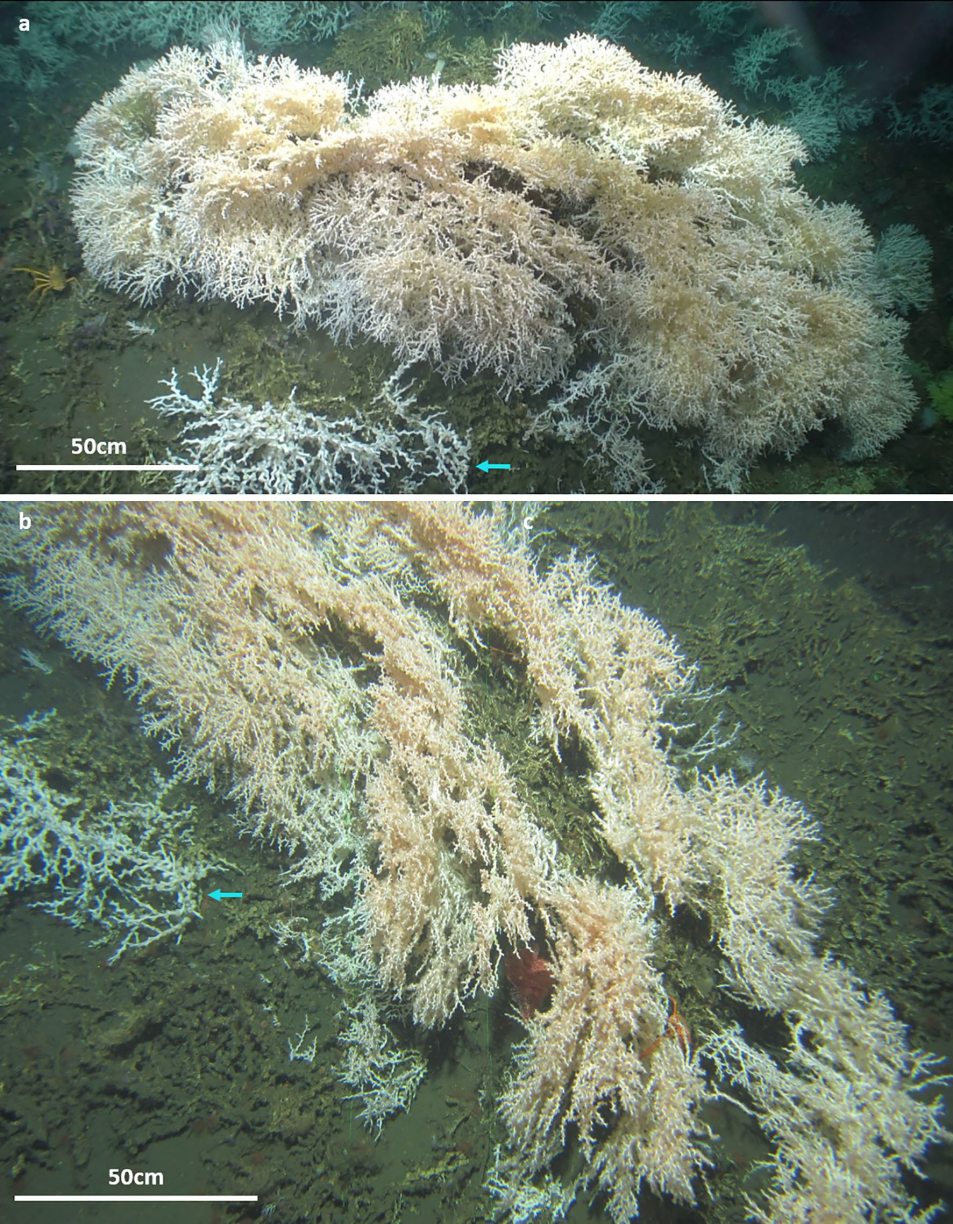
Extraordinary large *Madrepora oculata* framework of 1.2 m height observed on the Scary mound off Angola. (**a**) Panoramic view of the coral framework. (**b**) Zenithal view of the coral structure showing the different growing layers of the framework. Blue arrows indicate the *Lophelia pertusa* colonies in the vicinity of the large *M. oculata* framework. Credits: photographs by MARUM ROV SQUID.

**Figure 5:**
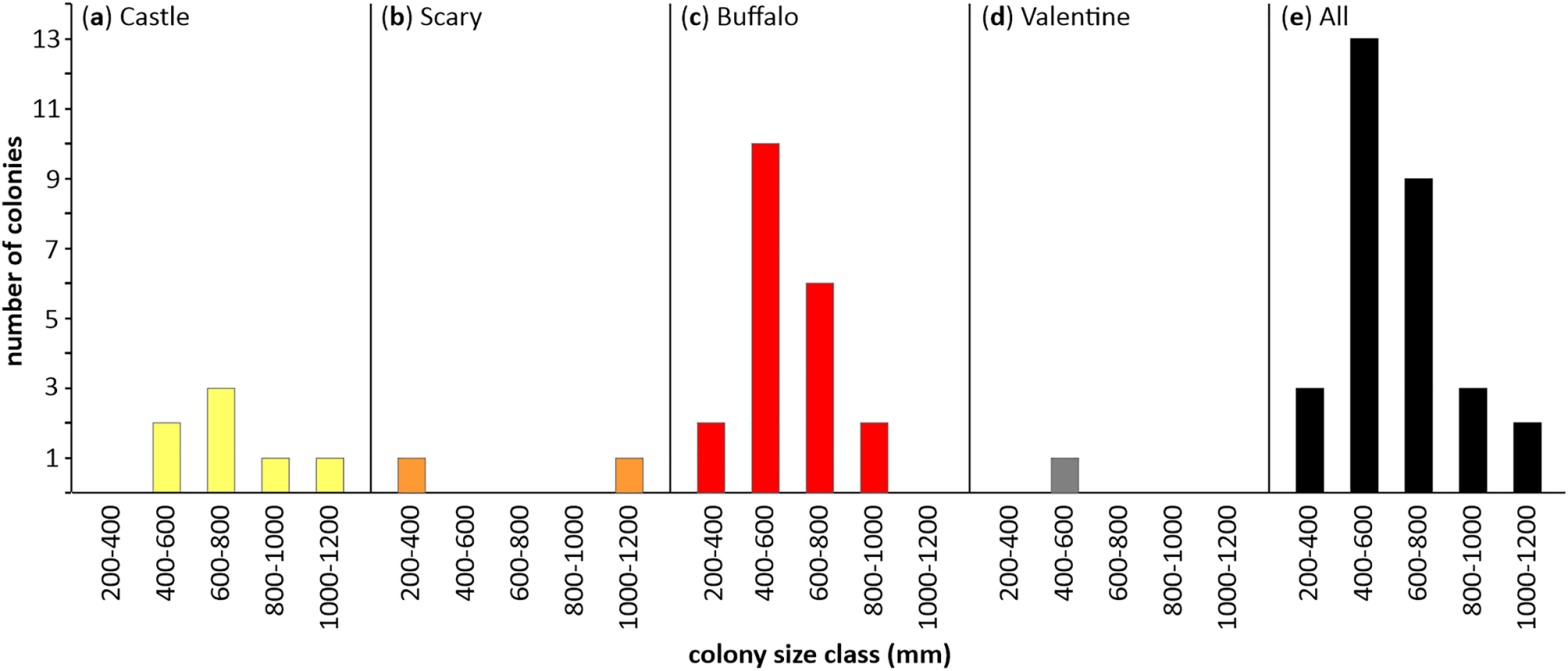
Coral colony size structure of the Angolan *Madrepora oculata* population. The number of *M. oculata* specimens for each defined colony size-class (given in mm) is depicted in this graph. They were documented for the Angolan mounds: (**a**) Castle, (**b**) Scary, (**c**) Buffalo, (**d**) Valentine, and (**e**) displays a compilation of all mounds.

An extraordinary large *M. oculata* framework was documented on Scary mound with a maximal height of 1250 mm (Figs. 3, 4). Two other remarkably large colonies were observed on Castle and Buffalo mounds with maximal heights of 1150 and 930 mm, respectively. Overall, the total height of the colonies varied between 250 and 1250 mm and colonies were on average 610 ± 210 (SD) mm high (Table 4). The scarce number of records from most locations did not allow for the performance of a proper analysis of the population structure. However, for Buffalo mound thirty-nine colonies were documented from which twenty *M. oculata*-colonies were suitable for measurements, most colonies were 400 to 600 mm high, but colonies of other size classes were also present. Sizes represented a typical Gauss distribution (Fig. 5). The alive/dead proportions of the measured colonies revealed similar length values for all colonies on all mounds with 170 ± 30 (SD) mm on average for the live part and 420 ± 170 (SD) mm for the dead part of the framework, respectively (Table 4).

**Table 4:** Measurements of total length and alive and dead proportions of *Madrepora oculata* colonies (R: range, Av.: average).

Coral mound	No. of colonies	Live part		Dead part		Total colony	
		R length (mm)	Av. length (mm ± SD)	R length (mm)	Av. length (mm ± SD)	R length (mm)	Av. length (mm ± SD)
Castle	7	80–460	200 ± 80	200–770	490 ± 20	370–1,150	700 ± 20
Scary	2	150–480	270	90–890	450	250–1,250	730
Buffalo	20	90–280	170 ± 70	70–170	390 ± 10	260–930	550 ± 10
Valentine	1	./.	220	./.	220	./.	440
All	30	80–480	170 ± 30	70–890	420 ± 170	250–1,250	610 ± 210

For each of the 30 colonies, 3–5 measurements were taken to cover the intra-colonial variability; here the minimum and maximum values (displayed as R length) of all measurements of each colony are provided.

### Estimation of *Madrepora oculata* colony ages

The estimated ages of the Angolan *M. oculata* colonies (considering available growth rates from the literature that vary between 3 and 18 mm year^−1^^33–35^) showed a range of 16 to 369 years and exhibited an average age of 95 ± 76 (SD) years (SD; Table 5).

**Table 5:** Age estimations for the Angolan *Madrepora* colonies based on growth rates being available for the Bay of Biscay, Mediterranean Sea and Norway.

Colony age estimation (years)	Bay of Biscay: 4.2 mm year^−1^ (1)	Mediterranean: 3 mm year^−1^ (2)	Mediterranean: 18 mm year^−1^ (2)	Norway: 11 mm year^−1^ (3)	Norway: 14 mm year^−1^ (3)	All geographical areas
Mean ± SD	143 ± 49	200 ± 68	33 ± 11	55 ± 19	43 ± 15	95 ± 76
Min	68	95	16	26	20	16
Max	264	369	62	101	79	369

For calculations, all *Madrepora* colonies measured on all mounds were considered (n = 30). Last column displays the mean age (± SD) as well as the minimum and maximum ages for all colonies measured considering all growth rates and geographical areas.

(1) Temperature: 10.0–11.9 °C^33^.

(2) Temperature: 12 °C^34^.

(3) Temperature: 6–9 °C^35^.

### Local environmental conditions

**Figure 6:**
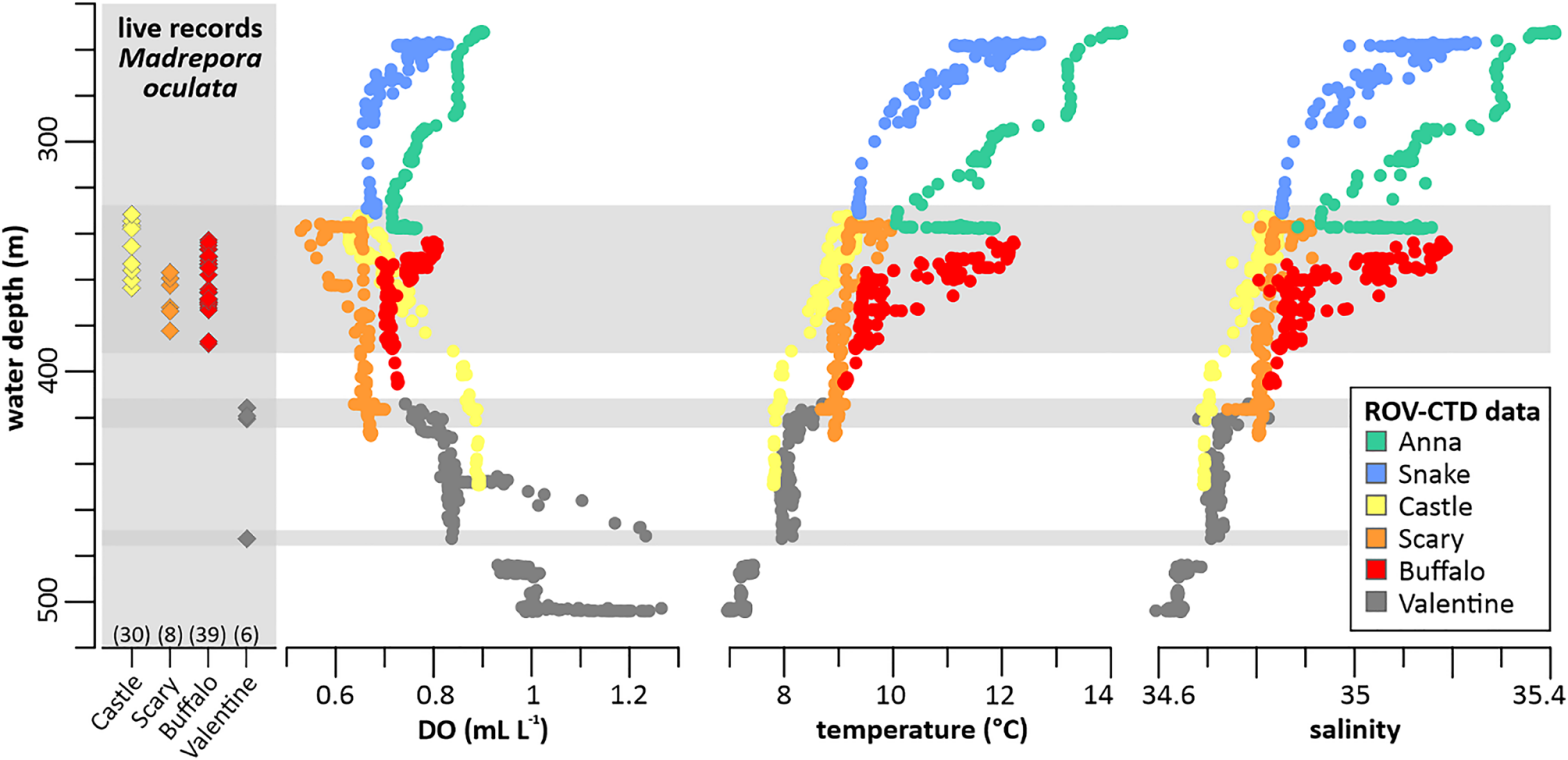
Depth distribution of *Madrepora oculata* and the corresponding environmental envelope off Angola. The water-mass properties (i.e. dissolved oxygen concentrations (DO), temperatures and salinities) were recorded with the ROV-mounted CTD during video surveys across various Angolan coral mounds (see legend for colour code; data for DO and temperatures were originally published in Hebbeln et al.^32^). Live colonies of *M. oculata* (diamond symbols) were only observed on Castle (n = 30), Scary (n = 8), Buffalo (n = 39), and Valentine (n = 6) mounds. Depth levels of live *M. oculata* occurrences are highlighted by horizontal grey bars.

Environmental parameters recorded by the ROV-CTD corresponding to the total depth range of living *M. oculata*-colonies (330–470 m) varied between 0.5 and 1.3 mL L^−1^ with regards to DO (range of mean values obtained for each of the four mounds (mean_range_): 0.7–0.9 mL L^−1^), 7.8 and 12.2 °C regarding the temperature (mean_range_: 8.1–10.3 °C), and between 34.6 and 35.2 in relation to salinity (mean_range_: 34.7–35.0; Table 3; Fig. 6). Interestingly, prior studies presented slightly larger ranges regarding DO (0.5 and 1.5 mL L^−1^) and temperatures (6.8–14.2 °C). These studies additionally incorporated repeated measurements by a conventional CTD through the water column and long-term measurements by benthic landers, and explained the higher variability in DO and temperature by the effect of internal waves and the larger geographical coverage^31,32^. With regards to the shallow mound sites with no *M. oculata* occurrence (Anna ridge, Snake mound), DO revealed similarly low values, while temperature and salinity were slightly higher than for the other mounds (Table 3; Fig. 6). In contrast, for the deepest part of Valentine mound (480–500 m), for which also no *M. oculata* occurrences were recorded, DO were slightly higher than for the other mounds, while temperature and salinities were significantly lower (Table 3).

## Discussion

Despite the rather extreme environmental setting along the Angolan margin with overall very low DO (minimum DO: 0.5 mL L^−1^) and rather high temperatures (maximum temperatures: 14.2 °C^31,32^), which, according to previous studies, is expected to cause severe stress on deep-sea benthic organisms such as CWCs^28,36^, vivid and large reef structures were found to be widely distributed there. The coral community in this location was dominated by *L. pertusa*^32^. However, even though, *M. oculata* was found in comparably low numbers of live and dead colonies (Fig. 2, Table 1), the average density of *M. oculata* thriving on the Angola coral mounds (0.53 ± 0.37 (SD) colonies m^−2^) was in the same order of magnitude as previously documented for the Mediterranean Sea (0.30 ± 1.14 (SD) to 0.81 colonies m^−2^^7,15^). However, it was lower than those reported for sites in the NE Atlantic (1.04 ± 0.80 (SD) to 1.64 ± 1.19 (SD) colonies m^−2^^4^). In contrast, the percental coverage data of *M. oculata* obtained for the Angolan coral mounds (0.29 to 1.74 ± 1.67 (SD) coverage m^−2^; Table 2) was clearly higher than the range of values previously documented for the NE Atlantic (0.01 ± 0.03 (SD) to 0.04 ± 0.04 (SD) coverage m^−2^^4^). This pattern might be related to the exceptional large colonies with heights of up to 1125 mm that were documented for the Angola coral mounds (Table 4). The reason for this different demographic pattern is unclear at this point. However, it is well known that recruitment (in plants) is a result of different processes like settlement, growth and mortality of seedlings^37^, which might also be applicable to coral larvae. Accordingly, we hypothesize that the *M. oculata* populations off Angola, comprising of few but large colonies, might experience less recruitment as reported for terrestrial forests. There, the presence of many small trees indicated ongoing recruitment, while forests with fewer but larger trees point to less intense recruitment^37^. Nevertheless, the population structure observed on Buffalo mound (where most *M. oculata* colonies were documented) indicates that the majority of the colonies had heights of 400–800 mm, while only a small amount of large (up to 930 mm in height) as well as of small colonies were present (Fig. 5). The low number of large colonies could be explained by the maximum size that colonies can reach due to physical constrains (e.g., maximal weight and height supported by the coral structure), whereas the low number of small colonies (recruits) might be a result of a so-called "demographic accident". This might be a result of highly variable larval supply recruitment, which results in a large number of individuals of the next size class (Bramanti pers. comm.). Such an event would explain why the *M. oculata* population on Buffalo mound appeared to be a growing population that has experienced a so called "recruitment accident" (Fig. 5).

Even if the overall low number of *M. oculata* recorded on the Angolan coral mounds avoids a robust statistical analysis, the depth-related environmental changes—in particular changes of DO and temperature measured with the CTD during the ROV video observations in the direct vicinity of the coral communities (Fig. 6; Table 3) - provide first insights into potential controls of their distribution and colony size spectrum. This might be an explanation for the observed intra-mound variability (Tables 2, 4). Regardless of the presence or absence of *M. oculata,* DO were very low ranging from 0.5 to 1.3 mL L^−1^ (Table 3) at all depths analysed (250–500 m). The majority of *M. oculata*-colonies (91%) as well as the largest colony sizes (930–1250 mm; Table 4) were documented between 330 and 390 m water depth (Castle, Scary, and Buffalo mounds), which correspond to DO of only 0.5 and 0.9 mL L^−1^ (Fig. 6, Tables 3, 4). Despite the moderate fluctuations, these values are by far the lowest DO previously observed for living CWCs (North Atlantic: ~ 1–2 mL L^−1^^18,29,38^). Nevertheless, they are slightly above values reported for an extinct coral population on the Namibia shelf, where DO of below 0.5 mL L^−1^ prevents a present-day coral colonization^31^ and even may have caused their local extinction about 4500 years ago^39^. However, the presence of vivid CWC reefs off Angola indicates that the prevailing low DO constitute no severe stressor per se and that the Angolan coral communities have adapted to such low oxygen levels^32^. It is assumed that the high amount and quality of food off Angola (illustrated by a high net primary production (NPP) of > 3400 g C m^−2^ day^−1^, see Table 6) counteract potential deleterious effects of hypoxia^31^. Additionally, aquaria experiments demonstrated that continuous food supply can neutralize or at least minimize the negative effects of some stressors on the corals´ metabolism allowing them to withstand extreme conditions^40,41^. A further explanation is offered by a recent study showing that *L. pertusa* lives in symbiosis with microbes that provide new nitrogen sources, compensating for losses caused by enhanced metabolic activity due to stressful environmental conditions^42^, such as the hypoxia off Angola.

**Table 6:** Environmental envelop of geographical areas where *Madrepora oculata* data on occurrence, density and/or coverage as well as growth rates have been published or are included in this work (Angola).

Region	DO (min–max) (mL L^−1^)	T (min–max) (°C)	S (min–max)	NPP (min–max) (g C m^−2^ day^−1^)	Data source
Norway^1^	6.4–6.7	6.4–6.9	35.1–35.2	421–686	(1, 2)
Iceland^2^	5.8–6.2	7.6	35.2	774	(1, 2)
Ireland^3^	5.2–5.4	6.2–9.8	35.1–35.4	834	(1, 2, 3)
Bay of Biscay^4^	4.9–5.4	9.0–11.4	35.5–35.7	530–872	(1, 2)
Mediterranean^5^	4.7	13.1	38.1	554	(1, 2)
Mauritania^6^	1.0–1.3	9.5–11.0	35.2–35.4	582–1899	(1, 4)
Angola	0.5–1.3	7.8–12.2	34.6–34.9	3431–6994	(1, 5, 6)

*DO* dissolved oxygen, *T* temperature, *S* salinity, *NPP* net primary production.

1: Norwegian mid-shelf break, Træna (~ 67° N, 11.1° E), Røst (~ 67.5° N, 9.4° W), and Sotbakken (70.8° N, 18.7° W) reefs; 2: southern Iceland slope, Hafadjúp, ~ 63.3° N, 19.6° W; 3: Rockall Trough, Logachev cold-water coral mounds, ~ 55.5° N, 15.6° W; 4: Croisic (46.4° N, 4.7° W), Guilvinic (46.9° N, 5.6° W), and Petite Sole (48.1° N, 8.8° W) canyons; 5: western Mediterranean Sea, Gulf of Lions (~ 42.5° N, 3.5° E); 6: Mauritanian cold-water coral mound province, ~ 20°–17° N, 17° W.

(1) NPP: http://data.guillaumemaze.org/ocean_productivity^45^.

(2) WOA: O_2_, T, S^46^.

(3) T, S^47^.

(4) O_2_, T, S^29^.

(5) O_2_, T, S^31^.

(6) This study.

In contrast to the DO, the temperature data recorded during the video surveys off Angola show some distinct variations through depth that correlate well with the occurrence and density patterns of *M. oculata*. For the depth interval 330–390 m, where the most abundant and largest colonies have been documented, mean temperatures range from 8.4 to 10.3 °C (Table 3). In contrast, temperatures are considerably higher (mean: 11–12 °C) at shallower depths (250–330 m, Anna ridge and Snake mound) and slightly lower (mean: < 8 °C) at deeper depths (> 470 m, deep part of Valentine mound), where no living specimens were documented (Tables 1, 2). Therefore, the observed rather narrow temperature window is seemingly most suitable for the Angolan *M. oculata* populations, and interestingly, it perfectly matches the preferred temperature range (8.5–10 °C) reported for living *M. oculata* occurrences in the North Atlantic^43^. Consequently, as the overall prevailing hypoxic conditions already pose a certain degree of stress to the Angolan CWCs, it is possible that *M. oculata* is not capable to compensate a further stressor, such as temperatures above or below its preferred temperature range.

A comparison of the environmental setting off Angola with environmental conditions prevailing in some Atlantic and Mediterranean reef sites, where *M. oculata* is present, shows a distinct trend regarding temperature, salinity, DO, and NPP (data compiled from various sources (^29,44–46^; see Table 6). *Madrepora oculata* thrives in a wide thermal envelope ranging from cold temperatures of ~ 6 °C at the most northern CWC sites off Norway and Ireland to ~ 12 °C off Angola, and even up to 13 °C in the Mediterranean Sea (Table 6). This remarkably wide thermal tolerance of *M. oculata* has previously been documented in aquaria experiments^25^. In contrast, salinities corresponding to occurrences of *M. oculata* in the Atlantic show rather small fluctuations and vary between 34.6 and 35.7, whereas the species tolerates values of up to 38.1 in the western Mediterranean Sea (Table 6). Even more impressive, *M. oculata* displays a wide (inter-regional) tolerance for DO as the species occurs under maximum values of 6.7 mL L^−1^ off Norway, but also under minimum values as low as 0.5 mL L^−1^ off Angola. The NPP data shows an opposite trend with the highest NPP corresponding to the southern upwelling areas off Mauritania and Angola (Table 6). On the one hand, this negative correlation between DO and NPP can be explained by the increased oxygen consumption due the remineralization of high fluxes of organic matter. On the other hand, the high availability of food might in turn compensate for the oxygen deficiency causing metabolic stress for the corals^31,32,40^. The interaction between DO (stressor) and food (compensator) has been assumed for the Angolan coral populations^31^, though aquaria experiments with *L. pertusa* reveal regionally dependent thresholds for DO^27,28^, which point to (a potentially genetic) regional adaptation^30^. This case exemplifies that we are still at the beginning of understanding how multiple factors control the proliferation of *M. oculata* (and other CWCs) and how local adaptation might enhance the complexity of the system. In this context, the estimated average age of 95 ± 76 (SD) years hints at fairly old *M. oculata* populations off Angola, which might underline that the species is capable to deal with the regional seasonal and even decadal environmental variability. However, it is questionable, if local adaptation capabilities of CWCs could keep pace with environmental changes related to global warming. Further, it should be taken into account that the ages presented here are only preliminary estimates and that growth rate measurements of Angolan specimens will be essential to confirm or readjust these age estimations.

**Figure 7:**
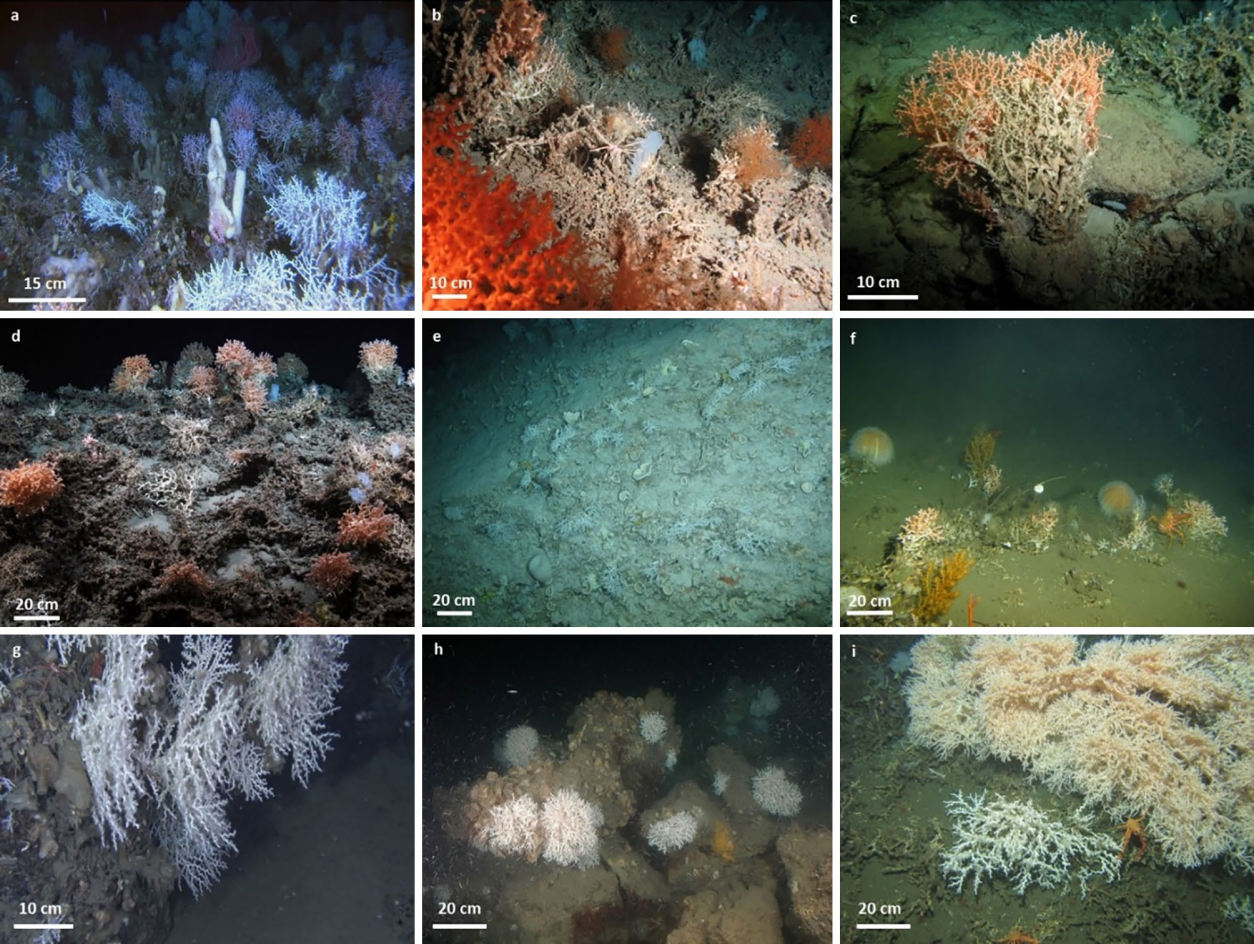
Variability of colony dimensions and morphotypes of *Madrepora oculata* documented for different regions in the Atlantic Ocean and the Mediterranean Sea. (**a**) off Norway (ca. 20 km southwest of Stornest off mid-Norwegian shelf break), 453 m depth, bushy morphotype, (**b**) off Ireland (Porcupine Seabight, Belgica coral mound province), 808 m depth, bushy morphotype, (**c**) Gulf of Mexico (West-Florida slope), 520 m depth, fan-shape morphotype, **(d)** Gulf of Biscay (Petit Sole canyon), 900 m depth, cauliflower morphotype, (**e**) off the U.S. (Florida Straits, west off Bimini), 468 m depth, bushy morphotype, (**f**) off Mauritania (Tamxat coral mound complex), 396 m depth, bushy morphotype, (**g**) western Mediterranean Sea (Gulf of Lions, Lacaze-Duthiers canyon), 350 m depth, fan-shape morphotype, (**h**) western Mediterranean Sea (Gulf of Lions, Cap de Creus canyon), 190 m depth, cauliflower morphotype, (**i**) off Angola (Scary mound), 335 m depth (this study), massive fan-shape morphotype. Credits: **(a)** Pål Buhl-Mortensen, Institute of Marine Research, Norway; **(b,c,e)** MARUM ROV CHEROKEE; **(d)** Ifremer, ROV Victor6000, BobEco2011; **(f)** Tomas Lundälv, Tjärnö Marine Laboratory, University of Gothenburg; **(g)** Dierk Hebbeln, MARUM; **(h)** JAGO, IFM-GEOMAR & ICM-CSIC; **(i)** MARUM ROV SQUID.

Interestingly, *M. oculata* exhibits partially similar growth rates in regions with very different environmental conditions such as the Norwegian shelf and the Mediterranean canyons^34,35^ (Table 6). On the contrary, these experimental studies also indicated a high variability in growth rates among specimens collected from the same region^34^. Therefore, the (potentially) wide range of ages estimated for the Angolan *M. oculata* colonies (16–369 years; Table 5) might simply reflect the high intraspecific growth rate variability. In addition, regionally different environmental conditions might be one of the reasons for the overall highly variable colony dimensions (varying from some branches of a couple of centimetres to colonies of > 1 m height; e.g.,^24,47^) and plasticity (displaying different morphotypes and tissue coloration) documented for *M. oculata* at various sites in the Atlantic and Mediterranean (Fig. 7). In order to understand the response of marine species to environmental changes, it is essential to understand how phenotypic as well as genotypic characteristics have been shaped by the dispersal-selection balance through space and time^48^. The high phenotypic plasticity of *M. oculata* (Fig. 7) and the scarce knowledge on its phenology and genetic characteristics^49^, makes it difficult to identify the regionally important environmental factors and to decipher their complex interplay favouring (or suppressing) the occurrence, growth and longevity of *M. oculata.*

The observed regional differences highlight the importance of considering the environment as it might well be the case that environmental differences along geographical gradients play an equally important role as the species eco-physiological limits documented in the experimental works. Indeed, environmental factors can act as important selective agents shaping genotype and phenotype composition of populations^50^. Consequently, genotype and phenotype adaptations must be taken into consideration to improve the understanding of the geographical as well as the bathymetric distribution and performance of marine organisms on both global and regional scales.

Finally, the expected ocean warming and deoxygenation are predicted to be two of the most important changes affecting CWCs^28,51^. Furthermore, predictions anticipating a decline of fluxes of particulate organic carbon from the surface waters^28,36^ will result in a lower energy supply for the CWCs and, thus, potentially reducing their capabilities to cope with increasing stress. The most recent habitat suitability model for the North Atlantic anticipates a reduction of 30% of suitable habitats for *M. oculata* with a northern shift in median latitudinal distributions (ranging from 1.9° to 4.6° in latitude), and a shift of median suitable depths towards deeper waters^3,28^. However, the geographical differences reflected by the variability of environmental parameters compiled for various Atlantic reef sites with the occurrence of *M. oculata* further highlight the importance to integrate potential regional adaptions of the species considered, which will be a future challenge for upcoming habitat suitability models.

## Methods

### ROV video surveys

During the RV Meteor cruise M122 (ANNA) in 2016, coral mounds with thriving reefs at their summits were discovered along the Angolan continental margin^52^. These mounds extended over a distance of ~ 30 nautical miles (nm) and were limited to water depths of ~ 250–500 m (Fig. 2). High-resolution mapping with the two multibeam echosounder systems KONGSBERG EM710 and EM122 (for technical details see^52^) revealed that their morphologies ranged from complex mound structures to long ridges with heights of up to 100 m (Fig. 2). The remotely operated vehicle (ROV) MARUM-SQUID (manufactured by SAAB Seaeye, UK) was utilized during the cruise M122 to explore the seafloor´s benthic communities colonizing the Angolan coral mounds. During this cruise, a total of seven dives (transects) were performed crossing six coral mounds (see Table 1, Fig. 2). The ROV was equipped with five video and still cameras (for technical details see^52^). Out of these, the main camera for video documentation was the Insite Pacific MiniZEUS MKII—a full HD camera with a resolution of 2.38 megapixels. A DSPL Wide-i SeaCam with a considerably lower resolution of 750 × 576 pixels was used as a downward-looking camera providing a full overview of the area underneath the vehicle. An Imenco Tigershark still camera acquired images at a resolution of 12 megapixels. For size measurements of organisms on the seafloor, the ROV was further equipped with two Imenco Dusky Shark line lasers, which have the capacity to project two parallel laser beams (lines) at a distance of 30 cm.

### Occurrence, density, coverage and size measurements of *Madrepora oculata* colonies based on ROV footage

The ROV video footage recorded by a MiniZEUS HD camera was visualized with the free software VLC to document and to further analyse the occurrence and distribution of *M. oculata* colonies on the coral mounds. In addition, screen shots were taken from the MiniZEUS video records, when the living and dead parts of the colonies were clearly visible and the laser beams were switched on for size calibration (Fig. 3). These screen shots were used to measure the dead and living parts of the individual colonies following the methodology of Vad et al.^53^ and using the free software Image J. For each measured colony (total number: 30), three to five repetitive measurements were taken to cover the intra-colonial variability.

The video frames recorded by the DSPL Wide-I SeaCam downward-looking camera were used to calculate species density and coverage of the seafloor as these provided (i) a complete coverage of the seafloor underneath the vehicle, and (ii) facilitated the calculation of the total area covered by using the two parallel laser beams as a scale. Downward-looking video frames appropriate for analyses were selected based on the detailed information regarding the colony locations documented in the MiniZEUS video footage. Despite the low resolution, it was possible to identify the colonies without difficulty as they stood out from the surrounding seafloor and other benthic organisms. *Madrepora oculata*-colonies were clearly visible in a total of 20 video frames, which were ultimately used to calculate the total area depicted in the video frame. The percentage of the area covered by *M. oculata*-colonies (expressed as coverage m^−2^) and the colony density (expressed as colonies m^2^) were determined by using the above-mentioned free software Image J. Due to the small number of colonies that could be measured (as a result of the limited number of suitable video frames), a statistical analysis regarding potential differences in occurrence among mounds was not feasible.

### Age estimations of *Madrepora oculata* colonies

In order to estimate an average age for the Angolan colonies, the minimum and maximum heights measured for 30 *M. oculata*-colonies and the growth rates published for this species in other regions, were used, following the methodology of Vad et al.^53^. Growth rate estimations for *M. oculata*, ranging between 3 and 18 mm year^−1^, are available for the Mediterranean Sea, the Gulf of Biscay and off Norway, and derive from aquaria and radio carbon-dated specimens^33–35^. As the general environmental setting in the tropical South Atlantic (hypoxic, warm and highly productive^31^) differs considerably from those in the temperate NE Atlantic and Mediterranean, all available growth rates were considered in order to perform the age estimates for the Angolan *M. oculata*-colonies. All colony measures were averaged and standard deviations were calculated. Furthermore, the minimum and maximum values for colony heights were taken into consideration for the age estimations.

### Data of water mass properties

During all video surveys, the ROV was additionally equipped with a CTD (Seabird SBE37) and water-mass properties (i.e. temperature, salinity and DO) were continuously recorded in the direct vicinity of the Angolan coral communities^52^. The ROV-CTD data have already been published to describe the occurrence of *L. pertusa* population thriving in the hypoxic and warm waters off Angolan^32^, and are used in this study to relate the distribution pattern of the *M. oculata* populations to variations of the hydrographic parameters. In addition, to comparing the Angolan environmental setting with environmental conditions of other CWC reef sites in the NE Atlantic (geographically stretching from Iceland to Mauritania) and the Mediterranean Sea, for which the occurrence of *M. oculata* is known, temperature, salinity and DO data were compiled which derived from published in situ measurements^29,46^ and/or the World Ocean Atlas (WOA13^45^). Moreover, data on the net primary productivity (NPP; applied here as a quantitative measure for the flux of organic material from the sea surface) were complemented for all regions used for comparison (derived from http://data.guillaumemaze.org/ocean_productivity^44^).

## Data Availability

The ROV-CTD-data are archived and can be retrieved at the World Data Center PANGAEA (10.1594/PANGAEA.904187). The ROV still images and screen captures as well as all the metadata on colony sizes, density, coverage and age analyses presented in this paper will also be archived in PANGAEA (https://www.pangaea.de).
